# Envelhecimento Vascular e Rigidez Arterial

**DOI:** 10.36660/abc.20210708

**Published:** 2022-10-05

**Authors:** Adriana Camargo Oliveira, Pedro Miguel Guimarães Marques Cunha, Priscila Valverde de Oliveria Vitorino, Ana Luiza Lima Souza, Gilcimar Divino Deus, Audes Feitosa, Eduardo Costa Duarte Barbosa, Marco Mota Gomes, Paulo Cesar B. Veiga Jardim, Weimar Kunz Sebba Barroso

**Affiliations:** 1 Universidade Federal de Goiás Liga de Hipertensão Goiânia GO Brasil Universidade Federal de Goiás , Liga de Hipertensão , Goiânia , GO – Brasil; 2 Universidade do Minho Escola de Medicina Braga Portugal Universidade do Minho Escola de Medicina , Braga , Portugal; 3 Pontifícia Universidade Católica de Goiás Escola de Ciências Sociais e da Saúde Goiânia GO Brasil Pontifícia Universidade Católica de Goiás - Escola de Ciências Sociais e da Saúde , Goiânia , GO – Brasil; 4 Universidade Federal de Goiás Programa de Pós-Graduação em Ciências da Saúde Goiânia GO Brasil Universidade Federal de Goiás – Programa de Pós-Graduação em Ciências da Saúde , Goiânia , GO – Brasil; 5 Pontifícia Universidade Católica de Goiás Escola de Ciências Exatas e da Computação Goiânia GO Brasil Pontifícia Universidade Católica de Goiás – Escola de Ciências Exatas e da Computação , Goiânia , GO – Brasil; 6 Universidade de Pernambuco Recife PE Brasil Universidade de Pernambuco , Recife , PE – Brasil; 7 Universidade Católica de Pernambuco Recife PE Brasil Universidade Católica de Pernambuco , Recife , PE – Brasil; 8 Complexo Hospitalar Santa Casa de Misericórdia de Porto Alegre Porto Alegre RS Brasil Complexo Hospitalar Santa Casa de Misericórdia de Porto Alegre – Cardiologia, Porto Alegre , RS – Brasil; 9 Centro Universitario CESMAC Hospital do Coração Maceió AL Brasil Centro Universitario CESMAC – Hospital do Coração , Maceió , AL – Brasil

**Keywords:** Rigidez Vascular, Hipertensão, Fatores de Risco de Doenças Cardíacas, Análise de Onda de Pulso, Remodelação Vascular

## Abstract

O envelhecimento biológico é reflexo da interação entre genética, idade cronológica e fatores externos; é a base para novos conceitos em envelhecimento vascular, cuja progressão é determinada pela diferença entre idade biológica e cronológica. Do ponto de vista estrutural, os efeitos do envelhecimento vascular são mais evidentes na camada média das grandes artérias elásticas e resultam em aumento da rigidez arterial, da dilatação do lúmen e da espessura da parede. Esses efeitos são descritos no *continuum* de envelhecimento cardiovascular (proposto por Dzau em 2010) em que as etapas progressivas de lesões da microvasculatura de coração, rins e cérebro, têm início a partir do processo de envelhecimento. O aumento da rigidez arterial pode ser verificado de forma não invasiva por vários métodos. Os eventos cardiovasculares têm sido tradicionalmente previstos utilizando escores que combinam fatores de risco convencionais para aterosclerose. No *continuum* cardiovascular clássico (Dzau, 2006), é desafiador avaliar o peso exato da contribuição de cada fator de risco; entretanto, por refletir o dano precoce e cumulativo desses fatores de riscos cardiovascular, a rigidez arterial reflete o verdadeiro dano à parede arterial. Este artigo fornece uma visão geral dos mecanismos da fisiopatogenia, alterações estruturais das artérias e consequências hemodinâmicas do envelhecimento arterial; métodos não invasivos para a avaliação da rigidez arterial e da medida central da pressão arterial; o *continuum* de envelhecimento cardiovascular, e aplicação do conceito de rigidez arterial na estratificação de risco cardiovascular.

## Fisiopatogenia do envelhecimento vascular

O envelhecimento é um dos principais fatores de risco para doenças e eventos cardiovasculares, principais causas de morte no mundo. ^1 - 3^ Entretanto, mais importante que a idade cronológica (tempo desde o nascimento), é a qualidade, a velocidade do envelhecimento e a forma como ele se reflete em anos livres de doença. ^3^

O envelhecimento sistêmico é reflexo não somente da idade cronológica, mas também do declínio na função fisiológica (idade biológica), impulsionado pela exposição crônica a baixos níveis de inflamação - “pró-inflamação”, contribuindo para senescência celular e envelhecimento patológico. Modificações pró-inflamatórias celulares e da matriz, associadas à idade, são a base para um envelhecimento vascular acelerado (EVA), em que a idade biológica supera a cronológica, com aumento exponencial na patogênese da hipertensão e aterosclerose, predispondo a doenças CVs e mortalidade precoces. ^3^

Com avançar da idade, o estresse físico, mental e ambiental aumenta devido às contínuas adaptações às mudanças nas circunstâncias de vida. O aumento do estresse desencadeia ativação neuroendócrina do sistema renina angiotensina aldosterona (SRAA), sistema nervoso simpático (SNS) e endotelina-1 (ET-1). Esses eventos de “sinalização pró-inflamatória” atuam nas células vasculares arteriais promovendo secreção e produção de citocinas e quimiocinas que se acumulam na parede arterial, como: proteína quimiotática de monócitos 1 (MCP-1), fator de transformação do crescimento beta 1 (TGF-β1), metaloproteinases da matriz (MMPs), calpaína-1 e glóbulo de gordura do leite - fator de crescimento epidérmico (MFG-E8), conhecidas como fenótipo secretor arterial associado à idade, bem como ativação ou inativação de fatores de transcrição (Ets-1, NF-κB, Nrf2 ou Sirt1). ^2 , 6 , 7^

As espécies reativas de oxigênio (ROS) estão aumentadas na parede arterial envelhecida, e sua principal fonte é a nicotinamida adenina dinucleotídeo fosfato (NADPH) oxidase. Os níveis das proteínas antioxidantes cobre-zinco superóxido dismutase (Cu/Zn SOD), SOD e SOD da matriz extracelular são regulados negativamente durante o envelhecimento. Este desequilíbrio, juntamente com aumento da angiotensina II e ET-1, aumenta a expressão de NADPH e a produção de ROS com consequente pró-inflamação, disfunção endotelial e enrijecimento da parede arterial envelhecida. ^2 , 3 , 7 - 11^

O óxido nítrico (NO) regula dilatação arterial, enrijecimento e inflamação com o envelhecimento. Na parede arterial, a expressão da NO sintase e NO estão diminuídas. Além disso, NO interage com ROS para gerar peroxinitrito (ONOO–), que reduz a biodisponibilidade do NO, prejudicando relaxamento do endotélio e aumentando vasoconstrição e pró-inflamação. ^2 , 3 , 7^

Essas alterações fenotípicas moleculares pró-inflamatórias eventualmente levam a alterações fenotípicas celulares e de matriz extracelular, devido ao estresse oxidativo e danos ao DNA, como senescência replicativa (redução dos telômeros e inativação da telomerase) e senescência prematura induzida por estresse (sem envolvimento dos telômeros). ^2 , 3 , 7^

As células arteriais sofrem diminuição da frequência mitótica, aumento no volume celular e encurtamento dos telômeros. A cascata de sinalização da angiotensina II leva redução da sinalização intracelular, autofagia funcional e aumento da produção de ROS. No nível celular, as células vasculares transformam-se em fenótipos heterogêneos: um subconjunto de células endoteliais e células musculares lisas vasculares torna-se senescente, enquanto outro torna-se mais proliferativo, invasivo/migratório, secretor e rígido. ^2 , 9^

A matriz extracelular sofre alterações como fibrose, elastólise, calcificação, amiloidose e glicoxidação. A fibrose desenvolve-se pelo aumento na produção e deposição de colágeno nas paredes arteriais, mediada pelas MMPs e TGF-β1, levando ao enrijecimento arterial. A elastólise ocorre devido à ruptura da rede de elastina interlamelar, pelas MMPs e elastase, resultando na diminuição da capacidade de armazenamento de energia elástica, complacência e resiliência arterial. Além disso, os produtos da elastólise participam da inflamação e calcificação arterial. No processo de calcificação, depósitos de cálcio na parede arterial aumentam devido à secreção de substratos semelhantes a ossos (como o colágeno II); ocorre elevada expressão de fosfatase alcalina (molécula pró-calcificação) e redução de moléculas anti-calcificação (osteonectina e osteopontina). Na amiloidose, as proteínas e fibrilas amilóides não compactadas aumentam na parede arterial, aumentando a rigidez e calcificação. Os produtos de glicação avançada encontram-se aumentados e contribuem para múltiplas alterações estruturais e funcionais no sistema arterial, como senescência, pró-inflamação e enrijecimento. ^2 , 7^

No nível tecidual, a pró-inflamação leva ao aumento do espessamento médio-intimal arterial, da disfunção endotelial, do enrijecimento arterial e da pressão arterial. Essas alterações compreendem a “síndrome da rigidez arterial pró-inflamatória”. ^2 , 6 , 7^

## Envelhecimento vascular – alterações estruturais das artérias

O efeito da idade é mais evidente nas grandes artérias elásticas. Principais alterações incluem aumento da rigidez (diminuição da distensibilidade), do diâmetro do lúmen e da espessura médio-intimal da parede. ^7 , 12 - 14^

A estrutura da árvore arterial consiste em três partes. Aorta, mais elástica, é a parte mais proximal e maior; as artérias musculares são a parte intermediária e as arteríolas são a parte mais distal e menor. Toda árvore arterial atua como conduto (distribuindo sangue do coração para capilares) e como amortecedor (alterando fluxo pulsátil gerado pela contração cardíaca intermitente em fluxo constante). Diferentes partes da árvore arterial desempenham papéis diferentes, as grandes artérias elásticas funcionam como amortecedores, enquanto as pequenas artérias e arteríolas funcionam como condutores. Diferenças entre artérias predominantemente elásticas ou musculares implicam na forma como reagem ao processo de envelhecimento, às mudanças de volume e pressão, e a fatores aterogênicos. ^3 , 7 , 12 - 15^

A camada média da artéria é a principal responsável pelas propriedades distensíveis da parede vascular; consiste em fibras elásticas, células musculares lisas, fibras colágenas e substância fundamental. A mudança dependente da idade é explicada pelo “estresse cíclico”. A sucessão dos ciclos cardíacos provoca alterações estruturais nas artérias devido à contração cardíaca intermitente e acomodação das variações hemodinâmicas de pressão entre sístole e diástole. Esse estresse pulsátil leva à desorganização da camada média das grandes artérias elásticas, por meio do afinamento gradual, divisão, desgaste e fragmentação da elastina. ^7 , 9 , 13 , 16 - 19^ Observa-se substituição desse material elástico por colágeno e formação de uma matriz mais rígida, com diferenciação osteogênica das células arteriais e calcificação. O processo resulta em enrijecimento da camada média pela transferência de estresse de fibras elásticas mais distensíveis para fibras colágenas menos distensíveis. ^7 , 12 , 13^

Essa degeneração é conhecida como “arteriosclerose” que deve ser diferenciado de “aterosclerose”, que afeta a íntima arterial, e não a média, por meio de um processo inflamatório endotelial com acúmulo de lipídios (estenose luminal). Embora essas duas lesões coexistam, a arteriosclerose tende a ser difusa nas artérias elásticas, enquanto as lesões ateroscleróticas são mais localizadas nas artérias elásticas e musculares suscetíveis (bifurcação carotídea e artérias coronárias). ^7 , 12 , 13^

Alterações estruturais nas grandes artérias devido à hipertensão são semelhantes às do envelhecimento (arteriosclerose), mas aparecem mais cedo, indicando que a hipertensão acelera o envelhecimento arterial. ^7 , 12 , 13^

As artérias musculares de tamanho médio dificilmente são afetadas pelo envelhecimento, pois são menos distensíveis que as artérias elásticas e, portanto, estão expostas a um estiramento cíclico muito menor. Nos jovens, as artérias são mais elásticas; com o avançar da idade, ocorre desaparecimento gradual da uniformidade elástica entre o sistema arterial proximal e distal, levando à diminuição progressiva da amplificação da pressão de pulso e prejuízo na interação ventricular-arterial. ^13 , 14 , 16 , 20^

A dilatação do lúmen ocorre após a degeneração e fratura da elastina, levando a uma parede arterial enfraquecida. A parede se torna mais rígida com a pressão de distensão, pois ocorre aumento da quantidade de fibras de colágeno. Assim, a relação entre tensão (pressão) e deformação (diâmetro) é não linear, com concavidade em direção ao eixo de distensão, de modo que há diminuição da distensão com o aumento da força. Esta propriedade é essencial para o funcionamento eficiente das artérias como condutos, de modo que, com a manutenção do estresse residual, os vasos não colapsam, garantindo o fluxo sanguíneo. A tensão da parede (T) equilibrada pela pressão transmural (P) e raio (r) (T = P · r, lei de Laplace) tem um único ponto de operação na curva pressão-diâmetro. O estresse na parede arterial torna-se ainda maior em consequência de um lúmen dilatado. Portanto, a dilatação e a degeneração arterial criam um ciclo vicioso que acelera ainda mais o envelhecimento vascular. ^7 , 12 - 14 , 19^

O aumento da espessura da parede depende da hiperplasia intimal. Os possíveis mecanismos responsáveis pelo espessamento da íntima incluem aterosclerose, elevação da pressão local e alterações bioquímicas com a idade. ^7 , 12 , 13^

Fatores de risco [hipertensão, tabagismo, excesso de sal, dislipidemia, diabetes, síndrome metabólica, doença renal crônica (DRC), inflamação, stress oxidativo, programação fetal e genética] podem potencializar processo de envelhecimento arterial fazendo com que o sistema vascular apresente, mais cedo que o esperado, características biológicas que levarão ao desenvolvimento de doenças CVs. ^1 , 10^

## Envelhecimento vascular: consequências hemodinâmicas

As artérias não exibem propriedades viscoelásticas uniformes e possuem mecanismos adaptativos. A elasticidade diminui das artérias proximais em direção as distais, enquanto a rigidez segue o caminho inverso. ^12 - 14 , 18^ Embora essa heterogeneidade tenha dificultado o desenvolvimento de modelos matemáticos capazes de avaliar a complacência arterial, outros modelos foram concebidos para explicar características hemodinâmicas da árvore arterial. ^12 , 14 , 18^

No modelo de Windkessel, o sistema arterial é comparado com carro de bombeiros, representando as grandes artérias como a cúpula de ar, as artérias de tamanho médio como a mangueira de incêndio e as pequenas arteríolas como o bocal. Assim, as artérias possuem duas características bem definidas: amortecimento (grandes artérias transformando fluxo pulsátil em fluxo constante para órgãos) e condução (pequenas artérias e arteríolas distribuindo o sangue do coração para órgãos). ^7 , 12^

O modelo de Windkessel possui limitações, pois assume que a velocidade de onda de pulso (VOP) tem valor infinito. Esse não poderia ser o caso, pois as funções de amortecedor e conduto não estão confinadas a artérias específicas, mas coexistem, levando à heterogeneidade da VOP ao longo da árvore arterial. Além disso, ocorre perda progressiva da função de amortecimento da aorta para as artérias periféricas mais musculares e rígidas, predominando a função de condução. Tal fenômeno de “reflexão de onda” ocasiona um aumento da amplitude da onda de pulso nos vasos do coração em direção a periferia, conhecida como pressão de amplificação. Além disso, a rigidez das artérias periféricas de tamanho médio é modulada pelo tônus vasomotor, dependente da função endotelial, SNS e SRAA.

Por isso, é melhor aplicar modelos propagativos ao sistema circulatório. Esses assumem que a VOP que viaja ao longo de determinada artéria tem valor finito. A equação de Moens-Korteweg: co¼p(Eh/2Rr), onde (co) representa velocidade da onda, (E) módulo de Young na direção circunferencial, (h) espessura da parede, (R) raio e (r) densidade de fluido derivou a equação: co¼p(V.dP/r.dV), onde (dV) é a mudança no volume arterial (V) e (dP) é a mudança na pressão que impulsiona a mudança no volume. Essa segunda equação é utilizada na pesquisa clínica e demonstra que a VOP está inversamente relacionada à distensibilidade do tubo arterial, expressa em dV/V.dP. A VOP fornece uma maneira direta de quantificar a rigidez arterial, quanto mais rígida a artéria, maior a VOP. ^7 , 12 , 14 , 17 , 18^

Assim, em vez do modelo de Windkessel, um modelo mais realista da árvore arterial seria o “modelo propagativo” constituído por tubo distensível simples que termina com resistência periférica, mas cujas propriedades elásticas distribuídas permitem geração de onda de pressão que percorre o tubo, no qual as funções de conduto e amortecedor são combinadas. A extremidade proximal do tubo corresponde à aorta e a distal às arteríolas de alta resistência. A onda de pressão gerada pela ejeção cardíaca viaja ao longo desse tubo da extremidade proximal à distal, onde essa onda direta é refletida de volta. ^7 , 12 , 14 , 17 , 18^

Tais modelos tornam possível explicar os fenômenos observados no sistema arterial real que não eram interpretáveis pelo modelo de Windkessel. Esses fenômenos incluem: uma onda de pressão secundária na diástole ou sístole tardia, e amplificação da pressão de pulso da aorta proximal para as artérias musculares distais, e explicam por que a rigidez arterial aumenta a pressão de pulso central e pressão arterial sistólica (PAS). Em indivíduos jovens ou adultos com envelhecimento arterial saudável, a onda retrógrada originada após a reflexão deve se sobrepor, e aumentar a pressão durante a diástole, e não durante a sístole, aumentando a perfusão coronária. ^7 , 13 , 14 , 18 , 19^

**Figura 1:**
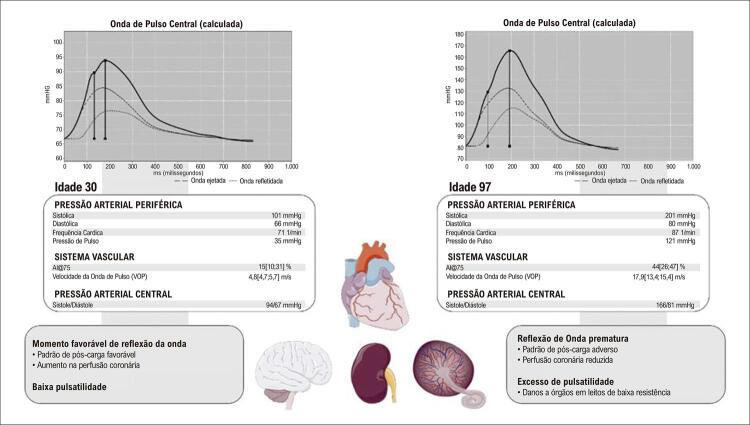
A rigidez arterial nas grandes artérias. Em adultos jovens saudáveis, uma aorta complacente (esquerda): 1) protege efetivamente o excesso de pulsatilidade causado pela ejeção ventricular esquerda intermitente; e 2) exibe uma velocidade de onda de pulso (VOP) mais lenta, permitindo que as ondas de pulso refletidas cheguem ao coração durante a diástole, aumentando a pressão de perfusão coronariana diastólica, mas não a pós-carga. Vários fatores, como envelhecimento e estilo de vida, aumentam a rigidez da parede aórtica, o que leva a várias consequências hemodinâmicas adversas. O enrijecimento aórtico leva ao aumento da impedância da raiz aórtica, com consequente aumento da amplitude da onda e chegada precoce de ondas refletidas ao coração. Essas alterações hemodinâmicas resultam em padrões adversos de carga pulsátil para o ventrículo esquerdo na sístole e redução da pressão de perfusão coronariana na diástole, em última análise, promovendo remodelamento miocárdico, disfunção e redução da reserva de perfusão (mesmo na ausência de doença coronariana epicárdica). Esse padrão hemodinâmico inverso também resulta em pulsatilidade excessiva na aorta, que é transmitida preferencialmente para leitos vasculares de baixa resistência (como rim, placenta e cérebro), pois nesses órgãos a pressão microvascular está mais diretamente associada às flutuações da pressão da arterial aórtica. Fonte: autores.

As ondas refletidas são originadas em vários locais, incluindo pontos de bifurcação das artérias de condução e pequenas artérias musculares. A vasoconstricção resulta em pontos de reflexão próximos ao coração, levando à formação precoce de ondas refletidas na aorta. O momento de chegada das ondas refletidas na aorta proximal depende da VOP dos vasos condutores. Além disso, o aumento da rigidez arterial, observado em idosos e hipertensos, promove uma chegada precoce da onda refletida, que viaja rapidamente ao longo da árvore arterial. Portanto, pequenas e grandes artérias contribuem para a reflexão precoce da onda, que retorna antecipadamente na sístole e se sobrepõe à onda de frente. Esse processo causa aumento na pressão arterial sistólica (PAS), e redução nas variações da pressão diastólica (PAD) e na pressão arterial ( Figura 1 ). ^7 , 13 , 14 , 19^

Uma onda de pressão que se propaga ao longo de tubo viscoelástico com numerosas ramificações é progressivamente amplificada do conduto central em direção distal devido às reflexões de onda e maior VOP em uma artéria periférica mais rígida. Como resultado a amplitude da onda de pressão é maior em artérias periféricas do nas centrais - “fenômeno de amplificação”. ^7 , 12 , 14 , 17 , 19 , 21^

## Métodos não invasivos para avaliação de rigidez arterial

A rigidez arterial pode ser avaliada em níveis sistêmico, local e regional. A análise sistêmica só pode ser realizada por modelos de circulação, enquanto a rigidez arterial local e regional podem ser medidas diretamente de forma não invasiva, tendo como vantagem que os parâmetros utilizados estão fortemente ligados à rigidez da parede arterial ( Tabela 1 ). ^7 , 14 , 18 , 19^

**Tabela 1:** Dispositivos e métodos usados para determinar a rigidez arterial regional, local e sistêmica

Ano da primeira publicação	Dispositivo	Método	Sítio da Medida	Valor preditivo para eventos CV (ano da primeira publicação)	Facilidade de utilização clínica
**Rigidez arterial regional**					
1984a	Complior®	Mecanotransdutor	Aorta, VOPcfb	Sim (1999)	++
1990a	Sphygmocor®	Tonometria	Aorta, VOPcfb	Sim (2011)	++
1991	WallTrack®	Echotracking	Aorta, VOPcfb	Não	+
1994	QKD	ECG +	Aorta, VOPcfb	Sim (2005)	++
1997a	Cardiovasc. Eng. Inc®	Tonometria	Aorta, VOPcfb	Sim (2010)	+
2002	Artlab®	Echotracking	Aorta, VOPcfb	Não	++
2002	Sistema de ultrassom	Sonda de Doppler	Aorta, VOPcfb	Sim (2002)	+
2002	Omron VP-1000®	Manguito de pressão	Aorta, VOPbtb	Sim (2005)	+++
2007	CAVI-Vasera®	ECG + manguito de pressão	Aorta, VOPctb	Sim (2014)	+++
2008	Arteriograph®	Manguito de pressão braquial	Aorta, VOPaab	Sim (2013)	++
2009	RMN, ArtFun®	RMN	Aorta, VOPaab	Sim (2014)	+
2010	Mobil-O-Graph®	Manguito de pressão braquial	Aorta, VOPcfc	Não	++
2010	Ultrafast®	Ecografia	Carótida comum	Não	–
2013	pOpmetre®	Pletismografia	Aorta, VOPdpb	Não	+++
2017	Withings®	Balistocardiografia	Aorta	Não	+++
**Rigidez arterial local**					
1991	WallTrack®	Echo-tracking	ACCd, ACF ^d^ , AB ^d^ .	Não	+
1992	NIUS®	Echo-tracking	AR ^d^	Não	+/–
2002	Artlab®, Mylab®	Echo-tracking	ACCd, ACF, AB	Sim (2014)	++
2017	Ultrasound systems	Echography	ACCd, ACF, AB	Não	+
2009	RMN, ArtFun®	RMN	AA ^d^ , AD ^d^	Não	+
**Rigidez arterial sistêmica**					
1989	Método de área	Decaimento diastólico		Não	+/–
1995	HDI PW CR-2000®	Windkessel modificado		Não	+
1997a	Cardiovasc. Eng. Inc®	Tonometria/Doppler/ Eco		Sim (2010)	+/–
2009	RMN, ArtFun®	RMN	AA, AD	Não	+

*^a^ Aparelho usado em estudos epidemiológicos pioneiros que mostram o valor preditivo da rigidez aórtica para eventos cardiovasculares; ^b^ VOP: velocidade da onda de pulso; cf: carótido-femoral; bt: braquial-tornozelo; ct: tornozelo cardíaco; aa: arco aórtico; dp: dedo da mão - pé. ^c^ Estimado, não medido; ^d^ Todas as artérias superficiais, incluindo particularmente aquelas mencionadas; Ao: aorta; ACC: artéria carótida comum; AFC: artéria femoral comum; AB: artéria braquial; AR: artéria radial; AA: aorta ascendente; AD: aorta descendente. Fonte: Adaptado de Laurent et al. (2019, p. 143-144)*

### Medida regional da rigidez arterial

A aorta é o vaso utilizado para determinação do enrijecimento arterial regional, pois as aortas torácica e abdominal são os maiores “amortecedores” da árvore arterial, e a VOP aórtica é preditor independente de desfechos CVs. ^22 - 32^

**Figura 2:**
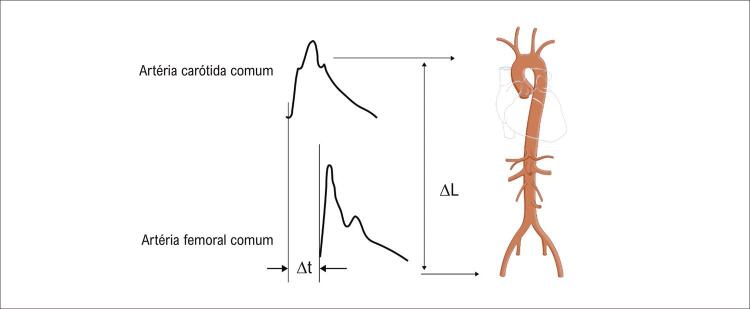
Medida da velocidade da onda de pulso carotídeo-femoral com o método “pé-a-pé”. Medida da velocidade da onda de pulso carotídeo-femoral com o método pé-pé. As formas de onda são geralmente obtidas por via transcutânea na artéria carótida comum direita e na artéria femoral direita. O atraso de tempo (Δt ou tempo de trânsito) é medido entre os pés das duas formas de onda. A distância (ΔL) percorrida pelas ondas é geralmente a distância da superfície entre os dois locais de registro, ou seja, a artéria carótida comum e a artéria femoral comum. A Velocidade de Onda de Pulso (VOP) é calculada como VOP = 0,8 × ΔL (m) / Δt (s). Fonte: autores

A medida da VOP carotídeo-femoral (VOPcf) é o método não invasivo, padrão-ouro para avaliação da rigidez arterial. Diversos estudos utilizaram a VOPcf e demonstraram que a rigidez arterial está relacionada a eventos CVs. ^7 , 14 , 19 , 29 , 30 , 33^ A VOPcf é medida de forma transcutânea (tonômetro), utilizando o método da velocidade “pé-pé” entre as ondas obtidas na artéria carótida direita e na femoral direita ( Figura 2 ). O “pé” da onda é definido no final da diástole, quando inicia a subida acentuada da frente de onda. A VOPcf é calculada pela fórmula: VOPcf (m/s) = *D* (metros) / ∆ *t* (segundos). A ( *D* ) pode ser calculada: (1) distância total medida entre os dois sítios (carótida e femoral); (2) subtraindo a distância do sítio carotídeo até a junção manúbrio-esternal da distância total ou (3) subtraindo a distância do sítio carotídeo até a junção manúbrio-esternal da distância da junção manúbrio-esternal até o sítio femoral. De todas as distâncias atualmente utilizadas, 80% da distância carótida-femoral direta (distância da artéria carótida comum até artéria femoral comum x 0,8) demonstrou ser mais precisa. ^3 , 13 , 14 , 19 , 32 , 33^

A medição da VOPcf por tonometria apresenta limitações como: a) o registro preciso da forma de onda de pressão femoral pode ser dificultado em pacientes com síndrome metabólica, obesidade e doença arterial periférica; b) presença de estenose aórtica, ilíaca ou femoral proximal, pode atenuar e atrasar a onda e c) a obesidade abdominal, especialmente em homens, e busto volumoso em mulheres podem influenciar na acurácia da distância medida. ^3 , 13 , 14 , 19 , 32 , 33^

Assim, a análise da VOP utilizando único local simplifica a medição. Foram desenvolvidos dispositivos que calculam a VOP em uma determinada via arterial a partir da análise da onda de pressão braquial obtida com manguito. Esses métodos incluem a determinação da diferença de tempo entre a onda Q no eletrocardiograma e os sons de Korotkoff no nível braquial. Arteriograph ^®^ estima VOP a partir de um manguito braquial em único ponto, usando método oscilométrico de determinação suprassistólica. Mobil-O-Graph ^®^ (Brasil, Dyna Mapa AOP ^®^ ) aproveita registros oscilométricos, obtidos por tripla aferição, da forma de onda da pressão da artéria braquial, no nível da PA média (calibração C1) ou diastólica (calibração C2), para compor onda de pulso aplicando função de transferência (algoritmo ARCSolver ^®^ ). Nesse último, a idade e a PA são usadas para refinar a estimativa da VOP. ^13 , 14 , 19 , 32 , 33^

Valores de referência para a VOPcf (tonometria) foram estabelecidos para indivíduos saudáveis e naqueles com fatores de risco CV em países europeus. ^34^ Ainda, valores de referência para o método oscilométrico, da pressão arterial sistólica central (PASc), índice de incremento aórtico (AIx) e VOP para indivíduos com e sem fatores de risco CV foram estabelecidos para a população brasileira ( Tabela 2 ). ^35^

**Tabela 2:** Valores de referência para pressão arterial central, velocidade da onda pulso e índice de amplificação aórtico (AIx) para homens e mulheres, com e sem fatores de risco cardiovasculares

Categoria da Idade	Sem Fatores de risco cardiovascular		Com Fatores de risco cardiovascular	

	Mulheres	Homens	Mulheres	Homens
**PASc**				
<30 anos	101 (90; 93; 113; 119)	113 (104; 109; 120; 123)	118 (102; 109; 127; 131)	123 (107; 114; 132; 144)
30-39 anos	109 (96; 102; 117; 123)	114 (102; 110; 121; 127)	120 (102; 110; 130; 143)	125 (108; 116; 133; 141)
40-49 anos	110 (99; 103; 117; 122)	116 (102; 109; 122; 126)	121 (104; 112; 134; 146)	123 (108; 115; 131; 141)
50-59 anos	110 (97; 104; 120; 124)	112 (100; 106; 1 18; 124)	124 (106; 114; 135; 146)	124 (105; 114; 134; 144)
60-69 anos	114 (100; 105; 120; 125)	112 (96; 101; 120; 127)	127 (105; 115; 141; 154)	123 (103; 112; 136; 149)
70+ anos	113 (100; 103; 121; 126)	116 (94; 104; 125; 129)	131 (108; 118; 146; 165)	125 (102; 111; 140; 156)
**PADc**				
<30 anos	73 (60; 66; 77; 85)	76 (66; 71; 82; 87)	82 (68; 73; 90; 97)	83 (72; 77; 93; 100)
30-39 anos	77 (67; 71; 83; 88)	80 (7 1; 75; 85; 88)	86 (71; 77; 95; 105)	88 (75; 80; 96; 103)
40-49 anos	79 (67; 73; 84; 88)	81 (74; 77; 86; 89)	86 (71; 78; 94; 103)	90 (75; 82; 97; 104)
50-59 anos	76 (64; 70; 82; 85)	82 (70; 77; 86; 88)	84 (71; 77; 92; 100)	88 (75; 80; 97; 103)
60-69 anos	76 (66; 71; 81; 87)	80 (68; 72; 83; 87)	81 (67; 74; 90; 98)	85 (71; 77; 93; 101)
70+ anos	76 (60; 70; 79; 83)	79 (60; 70; 84; 90)	81 (66; 72; 89; 97)	82 (68; 74; 91; 98)
**PPc**				
<30 anos	29 (23; 27; 37; 43)	36 (26; 32; 43; 53)	34 (24; 28; 41; 48)	38 (26; 31; 46; 52)
30-39 anos	30 (22; 26; 37; 44)	35 (25; 29; 42; 50)	34 (24; 28; 38; 46)	36 (25; 31; 41; 48)
40-49 anos	31 (22; 27; 36; 42)	32 (25; 28; 38; 45)	35 (25; 29; 43; 53)	33 (23; 28; 37; 46)
50-59 anos	34 (25; 28; 42; 49)	30 (25; 27; 35; 42)	39 (28; 32; 47; 58)	34 (25; 28; 41; 49)
60--09 anos	35 (28; 31; 43; 52)	31 (24; 28; 36; 49)	44 (30; 36; 55; 66)	37 (25; 31; 46; 58)
70+ anos	39 (28; 34; 45; 52)	37 (19; 27; 41; 51)	50 (33; 41; 63; 77)	42 (28; 34; 52; 66)
**VOP**				
<30 anos	4,9 (4,4;,4,5; 5,0; 5,3)	5,2 (4,9; 5,1; 5,4; 5,7)	5,3 (4,7; 5,0; 5,6; 6,0)	5,5 (5,0; 5,3; 5,8; 6,3)
30-39 anos	5,4 (5,0; 5,2; 5,8; 6,1)	5,7 (5,3; 5,5; 5,9; 6,1)	5,8 (5,3; 5,5; 6,2; 6,7)	6,1 (5,5; 5,8; 6,4; 6,7)
40-49 anos	6,4 (5,7; 6,0; 6,7; 6,9)	6,5 (5,9; 6,2; 6,8; 7,0)	6,8 (6,0; 6,4; 7,2; 7,7)	6,8 (6,2; 6,4; 7,1; 7,5)
50-59 anos	7,5 (6,7; 7,0; 7,8; 8,2)	7,4 (6,9; 7,2; 7,9; 8,0)	7,9 (7,1; 7,5; 8,3; 8,8)	7,9 (7,1; 7,5; 8,3; 8,7)
60-09 anos	8,9 (8,1; 8,5; 9,2; 9,4)	8,9 (8,2; 8,6; 9,1; 9,6)	9,3 (8,4; 8,8; 9,8; 10;4)	9,2 (8,4; 8,7; 9,7;10,2)
70+ anos	11,3 (10,2;10,4;12,5; 13;2)	11.0 (10,1; 10,6; 11,6; 12,3)	11,8 (10,2; 10,8; 12,9; 14,0)	11,2 (9,9; 10,4; 12,1; 13,2)
**AIx**				
<30 anos	20 (11; 13; 27; 33)	16 (4; 10; 23; 27)	28 (11; 20; 34; 38)	16 (2; 8;23; 30)
30-39 anos	22 (12; 16; 28; 34)	14 (1; 7; 18; 24)	26 (11; 18; 32; 37)	15 (3; 9; 2 1; 27)
40-49 anos	23 (9; 15; 29; 35)	15 (0; 6; 21; 25)	25 (10; 17; 34; 38)	15 (2; 8; 23; 30)
50-59 anos	22 (7; 12; 33; 39)	12 (2; 4; 19; 22)	24 (8; 14; 33; 39)	15 (3; 7; 24; 32)
60--09 anos	23 (9; 1 4; 34; 42)	17 (1; 5; 27; 43)	28 (11; 18; 37; 44)	1 7 (3;9; 26; 34)
70+ anos	28 (11; 20; 39; 42)	22 (5; 10; 33; 41)	33 (17; 25; 42; 48)	22 (4; 12; 31; 41)

PASc: pressão arterial sistólica central; PADc: pressão arterial diastólica central; PPc: pressão de pulso central; VOP: velocidade da onda de pulso e AIx: índice de aumentação. * Valores indicados como 50º (10º. 25º. 75º. E 90º) pontos percentuais. †Número de mulheres e homens CVRF-No: <30 anos (n=50 e 80): 30-39 anos (n=134 e 70): 40-49 anos (n=114 e 55): 50-59 anos (n=121 e 67): 60-69 anos (n=80 e 38): 70+ anos (n=32 e 26). ‡ Número de mulheres e homens CVRF-Sim: <30 anos (n=94 e 152): 30-39 anos (n=240 e 297): 40-49 anos (n=418 e 385): 50-59 anos (n=827 e 638); 60-69 anos (n= 919 e 561): 70+ anos (n=671 e 430). § Fatores de risco cardiovascular CVRF. Fonte: Adaptado de Paiva et al. (2020).

Apesar de sua relevância na predição de eventos CVs e na estratificação de risco, na prática clínica, a VOP ainda é subutilizada. Um grupo europeu propôs um escore clínico, o SAGE, capaz de identificar indivíduos com prioridade para a avaliação da VOP considerando variáveis facilmente disponíveis: “S”, *systolic blood pressure* (pressão arterial sistólica, PAS), “A” *age* (idade), “G” *fasting plasma glucose* (glicemia de jejum) e “E” *estimated glomerular filtration rate* (taxa de filtração glomerular estimada pelo CKD-EPI). ^36^ Esse escore foi aplicado na população brasileira com método oscilométrico e identificou que hipertensos com SAGE ≥8 deveriam ser encaminhados para análise da rigidez arterial, devido alta probabilidade de VOP aumentada. ^36 - 38^

### Medida local da rigidez arterial

A rigidez arterial local pode ser avaliada diretamente usando ultrassonografia das artérias carótidas por *echo-tracking* de alta resolução. O método tem como vantagem alta precisão para determinar diâmetro na diástole e mudanças de curso no diâmetro, em comparação com análise clássica de vídeo-imagem. A ressonância nuclear magnética torácica permite determinação combinada da estrutura e da função cardíaca e aórtica com precisão anatômica indiscutível, mas ao custo de menor resolução espacial e temporal. No entanto, a maioria dos estudos fisiopatológicos e farmacológicos utilizou técnicas de ecotracking. ^14 , 19 , 32 , 33^

### Medida sistêmica da rigidez arterial

Metodologia fundamentada em circuito elétrico utilizando modelo de Windkessel modificado desenvolvido para determinar complacência capacitiva proximal e complacência oscilatória distal. A complacência arterial sistêmica também pode ser determinada usando o “método da área”, que requer a medição do fluxo sanguíneo aórtico (velocímetro em incisura supraesternal) e pressão motriz associada à tonometria de aplanação sobre a artéria carótida comum direita. Limitações teóricas, técnicas e práticas prejudicam sua aplicação generalizada em ambiente clínico. ^14 , 19 , 33^

### Medida da pressão arterial central

A forma da onda da PA deve ser analisada ao nível central (aorta ascendente) uma vez que representa verdadeira carga imposta ao coração, cérebro, rins e parede arterial. A abordagem mais utilizada é a tonometria da artéria radial, seguida pela aplicação de função de transferência (SphygmoCor, AtCor, Sydney, Austrália) para calcular a forma de onda de pressão aórtica. A artéria radial, por ser sustentada por tecido ósseo, torna mais fácil a aplanação. ^7 , 14 , 19 , 32 , 33^

A onda de pressão aórtica pode ser estimada por tonometria das artérias carotídeas comuns, que exige maior conhecimento técnico, mas não necessita de função de transferência, pois os sítios arteriais são muito próximos e as formas de onda semelhantes. Novos métodos têm sido desenvolvidos para determinar o valor da PASc usando segundo pico sistólico (PAS2) nas ondas de pressão radial ou braquial. A calibração externa é necessária, feita com a PAS e a PAD braquial para calibrar a tonometria da artéria radial, e em seguida com a pressão arterial média e PAD radial para calibrar as formas de onda da aorta ou carótida. ^7 , 14 , 19 , 32 , 33^

**Figura 3:**
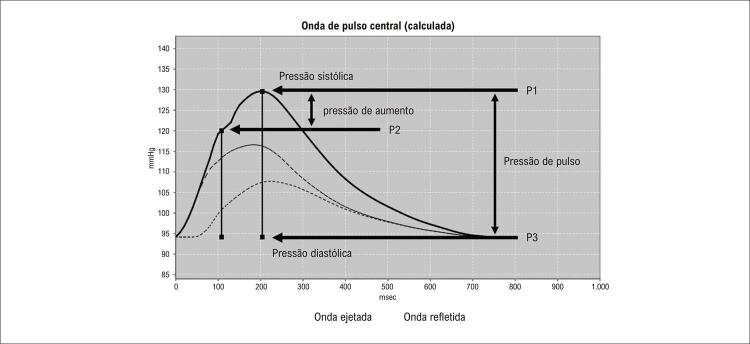
A forma da onda de pressão carotídea registrada por tonometria de aplanação. O fenômeno da reflexão da onda pode ser quantificado por meio do índice de aumento (AIx) - definido como a diferença entre o segundo (P2) e o primeiro (P1) picos sistólicos (P2 - P1 = PA, ou seja, pressão de aumento) expresso como uma porcentagem de PP (pressão de pulso), AIx = PA / PP. Fonte: autores.

A onda de pressão é composta pela onda de frente gerada pela contração ventricular e pela onda retrógrada, gerada pela reflexão nos sítios de bifurcação. Nos vasos elásticos, a VOP é baixa, e a onda refletida retorna à raiz da aorta durante a diástole. Na presença de rigidez arterial, a VOP aumenta e a onda refletida retorna precocemente, adicionando “aumento” durante a sístole. Este fenômeno pode ser quantificado pelo AIx, diferença entre segundo e primeiro pico sistólico (P2 – P1), em porcentagem, ( Figura 3 ). A idade e VOP são os principais determinantes do AIx. ^7 , 14 , 19 , 32 , 33^

Amplitude da onda de pressão nas artérias periféricas é maior que nas artérias centrais devido ao “fenômeno de amplificação”; portanto, a PAS periférica e a pressão de pulso braquial superestimam os valores da PAS e de pulso central em jovens. ^39^ A onda de pulso deve ser analisada por meio da pressão de pulso central (PPc), da PASc e do AIx. ^14 , 19 , 32 , 33^ Esses parâmetros são preditores independentes de mortalidade por todas as causas ^25 , 29 , 40^ e de eventos CVs. ^41 , 42^

Os valores de referência para PASc e AIx foram definidos para população europeia ^43^ utilizando o método tonométrico e para a população brasileira pelo método oscilométrico ^35^ ( Tabela 2 ).

A PASc, PPc, AIx e VOP não podem ser utilizados indistintamente como índices de rigidez arterial, pois são determinantes diferentes. A PASc, PPc e AIx dependem da VOP, da amplitude da onda refletida, do ponto de reflexão e da duração e padrão de ejeção ventricular, especialmente relacionadas às mudanças na frequência cardíaca (FC) e contratilidade ventricular. Condições fisiopatológicas e fármacos podem modificar PPc e AIx sem alterar VOP aórtica, sugerindo efeito predominante da onda refletida, da FC e da ejeção ventricular, e nenhuma mudança na rigidez aórtica. A influência da idade é maior no AIx do que na VOP antes dos 50 anos e maior na VOP que no AIx após essa idade. Portanto, a VOP, é uma medida direta da rigidez arterial, enquanto a PASc e o AIx são medidas indiretas. ^7 , 14 , 19 , 32 , 33^

## Rigidez arterial e o continuum cardiovascular

**Figura 4:**
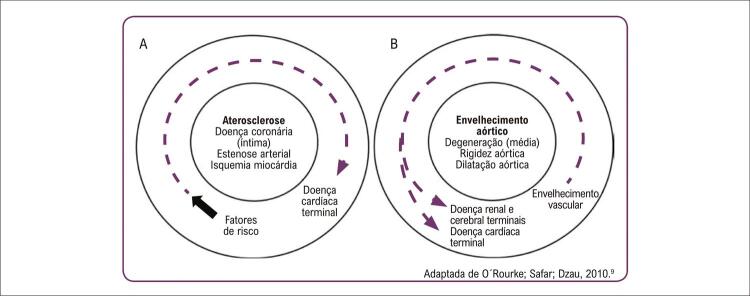
Comparação entre o continuum cardiovascular clássico (A) e o continuum do envelhecimento cardiovascular (B). Fonte: Barroso; Barbosa; Mota-Gomes, 2020.

A descrição clássica do *Continuum Cardiovascular,* publicada por Dzau et al. (2006) ^44^ descreve a progressão da doença CV ( Figura 4 ) fundamentada no processo de aterosclerose, que se inicia com exposição aos fatores de risco (hipertensão, diabetes, dislipidemia, tabagismo e obesidade), evoluindo em etapas que culminam na obstrução das artérias coronárias, isquemia e infarto do miocárdio, doença cardíaca terminal, insuficiência cardíaca e morte. Embora este modelo destaque aspectos fisiopatológicos relacionados a genes, moléculas, processos químicos e mecanismos intracelulares associados à aterosclerose, ignora contribuições do envelhecimento cardiovascular, derivado de alterações físicas e mecânicas das estruturas vasculares. ^3 , 45 , 46^

Em 2010, foi proposto novo modelo, o *Continuum do Envelhecimento Cardiovascular* ^46^ ( Figura 4 ), que tem como base o processo de arteriosclerose, e se inicia com o envelhecimento arterial, e progride para desenvolvimento de doença microvascular cardíaca, cerebral e renal terminal, incapacidade e morte. ^3 , 7 , 46^

Esta nova abordagem enfatiza a degeneração progressiva da aorta com consequentes efeitos nocivos para órgãos-alvo. A abordagem amplia as considerações da doença arterial para além daquelas causadas por obstrução e isquemia, para um progressivo enrijecimento das artérias elásticas que ocorre com o avançar da idade e se manifesta como aumento da VOP e AIx. ^3 , 7 , 46^ O aumento na VOP aórtica em 1 m/s associou-se a um incremento de 15% na mortalidade CV e por todas as causas. ^29^ Os parâmetros de PASc, VOP e o AIx demonstraram ser melhores preditores de risco CV e mortalidade do que a pressão arterial periférica. ^29 , 30^

**Figura 5:**
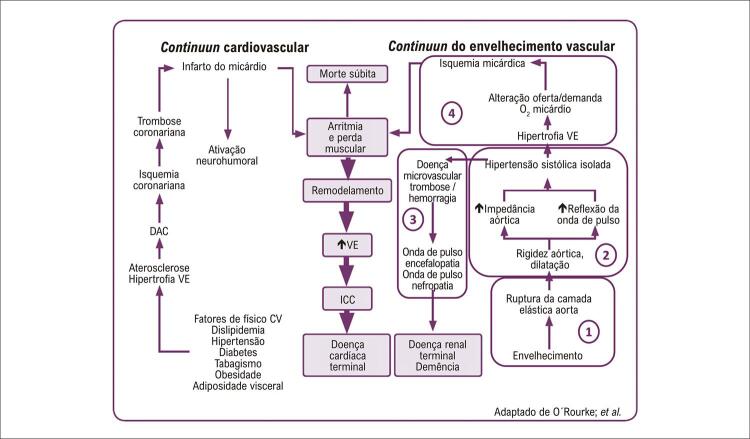
Associação do continuum cardiovascular clássico com o Continuum do envelhecimento cardiovascular; DAC: doença arterial coronariana; VE: ventrículo esquerdo; ICC: insuficiência cardíaca crônica. Fonte: Barroso; Barbosa; Mota-Gomes, 2020.

O *continuum* de envelhecimento CV é dividido em quatro estágios descritos a seguir: ( Figura 5 ). ^3 , 46^

- Estágio 1: Os batimentos cardíacos levam à fadiga e fratura das lamelas de elastina, com consequente dilatação da aorta e transferência do stress mecânico para fibras de colágeno, responsáveis pela rigidez arterial. ^3 , 46^
- Estágio 2: Enrijecimento aórtico leva a uma elevação da PAS, resultado tanto do aumento da rigidez da aorta proximal quanto do retorno precoce da onda refletida durante a sístole. Consequentemente, ocorrem aumento da pós-carga ventricular, hipertrofia ventricular esquerda (HVE), maior consumo de oxigênio pelo miocárdio e redução da perfusão coronária. ^3 , 46^
- Estágio 3: A contração cardíaca intermitente transmite o fluxo pulsátil para a aorta enrijecida (diminuição da capacidade de amortecimento) e se estende perifericamente para a microvasculatura, com aumento das forças de cisalhamento, especialmente nas pequenas artérias de órgãos com alto fluxo sanguíneo e baixa resistência microvascular (cérebro, rins, testículos, fígado e placenta). ^3 , 46^
- Estágio 4: o coração hipertrofiado contrai mais lentamente, de modo que a duração do período da sístole é aumentada e da diástole é reduzida em qualquer FC. Estas alterações comprometem fluxo sanguíneo coronário, que não consegue suprir a demanda devido à diminuição da pressão aórtica durante a diástole e do tempo da diástole. A combinação entre maior demanda de oxigênio e diminuição da capacidade de perfusão coronária predispõe à isquemia, independente do estreitamento coronário, que piora com aterosclerose. Surge então um ciclo vicioso: a isquemia causa maior comprometimento do relaxamento do ventrículo e prolongamento do tempo de ejeção, que consequentemente leva ao aparecimento de mais isquemia. ^3 , 46^

Os dois “contínuos” podem ser vistos independentemente, mas interagem no desenvolvimento da doença CV em estágio final. As vias finais são as mesmas, descrevendo complicações da isquemia miocárdica e a evolução para doença cardíaca terminal, como consequência do enrijecimento e estreitamento arterial. Os dois contínuos são combinados na Figura 5 para explicar os efeitos nocivos da doença aterosclerótica e do envelhecimento, à medida que progridem ao longo de anos e culminam nas doenças da velhice. ^46^ A insuficiência cardíaca é comumente associada a doença microvascular cerebral e renal, causando deterioração cognitiva e insuficiência renal. ^46^

O cérebro requer alto fluxo sanguíneo e baixa resistência arterial, sendo susceptível ao trauma microvascular pulsátil e à hipoperfusão, principalmente a substância branca, menos vascularizada e perfundida que a cinzenta. Alterações da perfusão cerebral devido ao aumento da pulsatilidade, levam a remodelamento microvascular e baixa oxigenação, com progressão do declínio cognitivo, demência, infarto subclínico e acidente vascular encefálico (AVE). ^7 , 47^

O rim exibe a maior taxa de fluxo sanguíneo e a menor resistência vascular quando comparado aos demais órgãos. Por isso, é suscetível ao trauma pelo fluxo pulsátil, que causa danos aos glomérulos, albuminúria e redução da taxa de filtração glomerular. A DRC também causa enrijecimento das grandes artérias devido ao desequilíbrio do metabolismo mineral ósseo (aumento de osteoprotegerina, fator de crescimento dos fibroblastos e citocinas inflamatórias) e maior calcificação dos vasos. A hiperatividade do SRAA e do SNA reduzem a eliminação do sódio, contribuindo com enrijecimento arterial. Em indivíduos com DRC, a VOP aumenta, particularmente nos diabéticos. A rigidez das grandes artérias prediz de forma independente maiores chances de eventos CVs em pacientes com DRC. ^7 , 25 , 26^

O envelhecimento leva ao enrijecimento arterial e modifica a microcirculação, ocasionando declínio da função cardíaca, cerebral e renal. É possível que o dano microvascular possa ser prevenido e/ou retardado com tratamento destinado a reduzir a rigidez arterial e reflexão da onda. ^7^

## Envelhecimento arterial e risco cardiovascular

Parte do risco CV residual em hipertensos tem sido relacionado ao processo de EVA. A detecção precoce permite proteção CV mais eficaz. Na fisiopatologia do desenvolvimento de doenças CVs, há uma interação bidirecional de EVA e hipertensão. ^1 , 10 , 45^

Fatores de risco clássicos são importantes para selecionar, avaliar e direcionar orientações de estilo de vida e terapia medicamentosa. No entanto, o risco de doença CV ainda representa desafio; apesar da prevenção e esforços de tratamento, há uma necessidade de novos modelos fisiopatológicos para melhor compreensão do risco CV e seu tratamento. ^3 , 45 , 50^

Foi demonstrado que a lesão de órgão-alvo, como HVE e aumento da microalbuminúria, representam etapa delimitadora entre fatores de risco e eventos CVs. ^45^ Além disso, rigidez arterial, aumento da VOP e aumento da PASc são preditores independentes de eventos CVs. ^29 , 30^ Estes são exemplos de um processo patológico subjacente, porque o aumento da VOP pode determinar o grau de HVE pelo aumento da reflexão da onda de pulso na artéria, PPc e pós-carga. ^7 , 19^

Sendo assim, a rigidez arterial é útil para melhor orientar as investigações clínicas em indivíduos com risco CV baixo e moderado. ^1 , 10^ Esses parâmetros, considerados “biomarcadores” arteriais, podem ser melhores preditores que a proteína C reativa de alta sensibilidade. ^32 , 45^ A adição da VOP durante a classificação de risco melhorou a previsão de risco (13% para risco de doenças CVs em 10 anos para risco intermediário). ^30^ Essa informação, quando adequadamente acessada e utilizada, pode evitar que pacientes com EVA, sejam erroneamente classificados como risco baixo ou moderado quando, na verdade, já apresentam alto risco. ^45 , 50^

## Perspectivas

O envelhecimento vascular é responsável pelo aumento do risco cardíaco residual e pela carga global de doença CV. Estudos são necessários para validação clínica dos desfechos CVs, comparações entre diferentes métodos de avaliação, e estudos de intervenção terapêutica mediados por redes de pesquisadores em envelhecimento vascular. É necessária a promoção de educação continuada e o uso das tecnologias em estratégias preventivas com objetivo de realçar a importância do papel do envelhecimento vascular e integrá-lo na tomada de decisões clínicas por médicos. ^3 , 50 , 51^

A ciência tenta avançar com o melhor entendimento e aplicabilidade clínica de biomarcadores capazes de identificar precocemente o dano vascular. O objetivo é aumentar a precisão na estratificação de risco CV em indivíduos considerados de risco baixo ou intermediário. ^32^ As avaliações da PASc e da rigidez arterial (VOP) ancoram-se em evidências robustas para identificar precocemente dano vascular, e identificar e reclassificar indivíduos inicialmente considerados como de risco baixo e intermediário, para um risco alto. ^30 , 45^ Além disso, os valores de VOP ≥10 m/s podem caracterizar a presença de lesão subclínica em órgão-alvo e o aumento na PASc é preditor do desenvolvimento de hipertensão arterial. ^7 , 19 , 30 , 52^ É possível que, à medida que novas evidências sejam produzidas no contexto da doença hipertensiva e das doenças CVs, esse método venha a ser mais fidedigno e seguro para ser incorporado à prática clínica, objetivando identificar ainda mais precocemente o dano vascular. ^50^ Esse tipo de abordagem, pensando na medicina de precisão, traz a possibilidade de uma prática médica mais personalizada, com maior assertividade nas decisões relacionadas à classificação e ao tratamento das doenças CVs. ^50^
