# Supplementary material for: Envelhecimento Vascular e Rigidez Arterial
Source: Arq Bras Cardiol. 2022 Oct 5;119(4):604–15. [Article in Portuguese] doi: 10.36660/abc.20210708 (PMC9563886; doi:10.36660/abc.20210708)
Supplement: Translation

# Translation: Vascular Aging and Arterial Stiffness

**Authors:** Adriana Camargo Oliveira, Pedro Miguel Guimarães Marques Cunha, Priscila Valverde de Oliveria Vitorino, Ana Luiza Lima Souza, Gilcimar Divino Deus, Audes Feitosa, Eduardo Costa Duarte Barbosa, Marco Mota Gomes, Paulo Cesar B. Veiga Jardim, Weimar Kunz Sebba Barroso

**Affiliations:** 1 Brazil Universidade Federal de Goiás, Liga de Hipertensão, Goiânia, GO – Brazil; 2 Portugal Universidade do Minho Escola de Medicina, Braga – Portugal; 3 Brazil Pontifícia Universidade Católica de Goiás – Escola de Ciências Sociais e da Saúde, Goiânia, GO – Brazil; 4 Brazil Universidade Federal de Goiás - Programa de Pós-Graduação em Ciências da Saúde, Goiânia, GO – Brazil; 5 Brazil Pontifícia Universidade Católica de Goiás – Escola de Ciências Exatas e da Computação, Goiânia, GO – Brazil; 6 Brazil Universidade de Pernambuco, Recife, PE – Brazil; 7 Brazil Universidade Católica de Pernambuco, Recife, PE – Brazil; 8 Brazil Complexo Hospitalar Santa Casa de Misericórdia de Porto Alegre – Cardiologia, Porto Alegre, RS – Brazil; 9 Brazil Centro Universitario CESMAC - Hospital do Coração, Maceió, AL – Brazil

**Keywords:** Vascular Stiffness, Hypertension, Heart Disease Risk Factors, Pulse Wave Analysis, Vascular Remodeling

## Abstract

Biological aging occurs as a result of the interaction between genetics, chronological age and external factors. It is the basis for new concepts of vascular aging, whose progression is determined by the difference between biological and chronological age. From the structural point of view, the effects of vascular aging are more evident in the tunica media of large elastic arteries, marked by increased arterial stiffness, lumen dilation and wall thickness. These effects are described in the continuum of cardiovascular aging (proposed by Dzau in 2010), in which the progressive steps of microvasculature lesions of the heart, kidney and brain are initiated from the aging process. The increase of arterial stiffness can be detected by several non-invasive methods. Cardiovascular events have been traditionally described using scores that combine conventional risk factors for atherosclerosis. In the classic cardiovascular continuum (Dzau, 2006), to determine the exact contribution of each risk factor is challenging; however, since arterial stiffness reflects both early and cumulative damage of these cardiovascular risk factors, it is an indicator of the actual damage to the arterial wall. This article provides a general overview of pathophysiological mechanisms, arterial structural changes, and hemodynamic consequences of arterial stiffness; non-invasive methods for the assessment of arterial stiffness and of central blood pressure; the cardiovascular aging continuum, and the application of arterial stiffness in cardiovascular risk stratification.

## Physiopathology of vascular aging

Aging is one of the main risk factors for cardiovascular diseases (CVD) and events, the main cause of death in the world.^1 - 3^ More important than chronological age (passage of time from birth), however, is the quality and velocity of aging and how it is reflected in disease-free years.^3^

Systemic aging reflects not only the chronological age, but also the decline in physiological function (biological age), promoted by chronic exposure to low inflammation – “pro-inflammation”, contributing to cellular senescence and pathological aging. Matrix-degrading and pro-inflammatory cellular changes, associated with aging, are the basis for the accelerated vascular aging (AVA), in which biological age surpasses the chronological one, with an exponential increase in the pathogenesis of hypertension and atherosclerosis, predisposing to CVD and early mortality.^3 - 5^

With aging, physical, mental, and environmental stress increase due to the need for continuous adaptation to life changes. The increase in stress triggers neuroendocrine activation of the renin-angiotensin-aldosterone system (RAAS), sympathetic nervous system (ANS) and endothelin-1 (ET-1). These events are “pro-inflammatory signals” that act on vascular cells promoting the production and secretion of cytokines and chemokines that accumulate on the arterial wall, such as: monocyte chemoattractant protein-1 (MCP-1), transforming growth factor beta (TGF-β), matrix metalloproteinases (MMPs), calpain-1, and milk fat globule-epidermal growth factor 8 (MFG-E8), known as age-associated arterial secretory phenotypes, as well as activation or inactivation of transcription factors (Ets-1, NF-κB, Nrf2 or Sirt1).^2 , 6 , 7^

Reactive oxygen species (ROS) are increased in aged arterial wall, nicotinamide adenine dinucleotide phosphate oxidase (NADPH). The levels of the antioxidant proteins copper-zinc superoxide dismutase (Cu/Zn SOD), SOD, and extracellular SOD are negatively regulated during aging. This imbalance, combined with the increase in angiotensin II and ET-1 levels, increases NADPH expression and ROS production, with consequent pro-inflammation, endothelial dysfunction, and stiffening of the aged wall.^2 , 3 , 7 - 11^

Nitric oxide (NO) regulates aging-associated arterial dilation, stiffening and inflammation. In the arterial wall, the expression of both NO synthase and NO are decreased. In addition, NO interacts with ROS to generate peroxynitrite (ONOO –), which reduces NO bioavailability, affecting endothelial relaxation and increasing vasoconstriction and pro-inflammation.^2 , 3 , 7^

These proinflammatory molecular phenotype alterations eventually lead to cellular and matrix phenotypic changes due to oxidative stress and DNA damage, like replicative senescence (shortened telomeres and inactivation of telomerase) and stress-induced premature senescence (without telomere involvement).^2 , 3 , 7^

In arterial cells, mitotic rate is reduced, which is accompanied by cell volume increase and telomere shortening. The angiotensin II signaling cascade leads to intracellular signaling inhibition, functional autophagy, and increased ROS production. At cellular level, vascular cells develop several phenotypes – a subgroup of endothelial cells and vascular smooth muscle cells become senescent, and another subgroup become more proliferative, invasive/migratory, secretory, and rigid.^2 , 9^

Changes in the extracellular matrix occur, including fibrosis, elastolysis, calcification, amyloidosis and glucose oxidation, increased collagen synthesis and deposition in the arterial walls, mediated by MMPs and TGF-β1, leading to arterial stiffening. Elastolysis occurs due to rupture of interlamellar elastin fibers by MMPs and elastase, resulting in decreased arterial elastic energy storage, complacence, and resilience. Besides, the products of elastolysis seem to be involved in the arterial inflammation and calcification processes. Calcium deposits increase in the arterial wall due to secretion of bone-like substrate (like collagen II). In parallel, there is an overexpression of alkaline phosphatase (pro-calcification molecule) and reduction of anti-calcification molecules (osteonectin and osteopontin). In amyloidosis, increased uncompacted amyloid proteins and fibrils in the arterial wall causes arterial stiffening and calcification. The products of advanced glycation are elevated and contribute to several structural and functional changes in the arterial system, including senescence, pro-inflammation and stiffening.^2 , 7 - 9^

At tissue level, pro-inflammation leads to an increase of intima media thickness, endothelial dysfunction, and arterial stiffening and blood pressure. These changes form the basis for the proinflammatory arterial stiffness syndrome.^2 , 6 , 7^

## Vascular Aging – Arterial Structural Changes

The effects of age are more evident in large elastic arteries. The main changes include the increase of arterial wall stiffness (reduced distensibility), lumen diameter, and intima media thickness.^7 , 12 - 14^

The structure of the arterial tree consists of three parts. The aorta, the most elastic segment, is the most proximal and largest portion; the intermediate segment is composed of muscle arteries, and arterioles are the smallest and most distal portion. The arterial tree act as both a conduit (distributing blood from the heart to the capillaries) and cushion (changing pulsatile flow generated by cardiac intermittent contraction to steady flow). Different parts of the arterial tree play different roles; while large elastic arteries act as a cushion, arterioles work as conduits. Differences between predominantly elastic and muscular arteries influence their responses to aging process, to volume and pressure changes and to atherogenic factors.^3 , 7 , 12 - 15^

The tunica media is the main responsible for the distensibility of the vascular wall and consists of elastic fibers, smooth muscle cells, collagen fibers and fundamental substance. Age-dependent change is explained by the “cyclic stress”. The succession of cardiac cycles causes structural changes in the arteries because of intermittent cardiac contraction and hemodynamic pressure changes between systole and diastole. This pulsatile stress leads to disorganization of the tunica media of large elastic arteries, through the gradual thinning, division, deterioration, and fragmentation of elastin.^7 , 9 , 13 , 16 - 19^ This elastic material is replaced by collagen, with formation of a rigid matrix, osteogenic differentiation of arterial cells and calcification. The process results in stiffening of the tunica media by transference of the stress from more distensible elastic fibers to less distensible collagen fibers.^7 , 12 , 13^

This degeneration is known as “arteriosclerosis”, which should be differentiated from “atherosclerosis”, that affects the tunica intima, rather than the tunica media, through an endothelial inflammatory process with lipid accumulation (luminal stenosis). Although the two lesions coexist, arteriosclerosis tend to be more diffuse in the elastic arteries, while the atherosclerotic lesions are more located in susceptible elastic and muscular arteries (carotid bifurcation and coronary arteries).^7 , 12 , 13^

Structural changes in large arteries due to hypertension are similar to aging-related changes (arteriosclerosis), but have an earlier onset, indicating that hypertension accelerates arterial aging.^7 , 12 , 13^

Medium-sized muscular arteries are hardly affected by aging as they are less distensible than elastic arteries and are hence exposed to lower cyclic stretching. In young individuals, arteries are more elastic; with aging, there is a gradual disappearance of elastic uniformity between proximal and distal arterial system, leading to progressive decrease in pulse pressure amplification and negatively affecting the ventricular-arterial interaction.^13 , 14 , 16 , 20^

Lumen dilatation occurs after elastin fracture and degeneration, resulting in weakening of the arterial wall. The arterial wall becomes stiffer with the distension pressure, due to the increase of collagen fibers. Thus, there is a non-linear relationship between tension (pressure) and deformation (diameter), with concavity toward the distension axis, such that distension decreases as force increases. This is essential for an effective functioning of the arteries as conduits, with maintenance of a residual stress, without artery collapse, promoting good blow flow. Wall tension (T), balanced by transmural pressure (P) and radius (r) (T = P · r, law of Laplace) has a unique operating point in the pressure-diameter curve. The arterial wall stress is even greater as a consequence of a dilated lumen. Therefore, arterial dilatation and degeneration generates a vicious cycle that contributes to acceleration of vascular aging.^7 , 12 - 14 , 19^

The increase in wall thickness depends on intimal hyperplasia. The possible mechanisms responsible for increased intima–media thickness include atherosclerosis, elevation of local pressure, and biochemical changes with age.^7 , 12 , 13^

Risk factors – hypertension, smoking, excess salt consumption, dyslipidemia, diabetes, metabolic syndrome chronic kidney disease (CKD), inflammation, oxidative stress, fetal and genetic programming, can potentiate the process of arterial aging and the early development of biological features by the vascular system that will lead to the development of CVD.^1 , 10^

## Vascular Aging: Hemodynamic Consequences

Arteries do not have uniform viscoelastic properties and exhibit adaptative mechanisms. While elasticity decreases from the proximal to the distal arteries, stiffness follows the opposite pattern.^12 - 14 , 18^ Although such heterogeneity made it difficult to develop mathematical models to assess arterial compliance, other models have been constructed to explain hemodynamic characteristics of the arterial tree.^12 , 14 , 18^

In the Windkessel model, the arterial system is compared to a fire truck, in which large vessels would represent the air chamber, medium-sized arteries the fire hose and small arterioles the spout. Therefore, the arteries have two well-defined characteristics - the cushioning function (large arteries transforming pulsatile flow into constant flow to the organ) and the conduit function (small arteries and arterioles delivering blood from heart to organs and tissues).^7 , 12 - 14 , 18 , 19^

The Windkessel model has limitations, as it assumes an infinite pulse wave velocity (PWV). This could not be the case, because both conduit and cushioning functions are not limited to specific arteries, but rather coexist, leading to the heterogeneity of PWV. Besides, there is a progressive loss of the cushioning function from the aorta to the more muscular and rigid peripheral arteries, and a predominant conduit function. This phenomenon, of “wave reflection”, leads to an increase in the amplitude of the pressure wave from the heart vessels to the periphery, known as pressure amplification. In addition, the stiffness of medium-sized peripheral arteries is modulated by the vasomotor tone, depending on the endothelial function, the ANS and RAAS.

For this reason, it is better to apply propagative models to the circulatory system. These assume that PWV that travels along an artery has a finite value. The Moens–Korteweg equation: co¼p(Eh/2Rr), where “co” represents wave speed, “E” the Young’s modulus in the circumferential direction, “h” the wall thickness, “R” the radius, and “r” the density of fluid] derived the equation: co¼p(V.dP/r.dV), where dV is the change in arterial volume (V) and dP is the change in pressure driving the change in volume. This second equation is currently widely used in the clinical research and demonstrates that the PWV is inversely related to the distensibility of the arterial tube, expressed asdV/V.dP. The PWV provides a straightforward way to quantify arterial stiffness; the stiffer the artery the higher the PWV.^7 , 12 , 14 , 17 , 18^

Thus, rather than the Windkessel model, a more realistic model of the arterial tree would be a “propagative model” consisting of a simple distensible tube which terminates at the peripheral resistance. However, its elastic properties allow the generation of a pressure wave which travels along the tube, in which conduit and cushioning functions are combined. The proximal end of the tube corresponds to the central aorta, and the distal end to the high-resistance arterioles. The pressure wave generated by cardiac ejection travels along this tube from the proximal end to the distal end, where this forward wave is reflected back.^7 , 12 , 14 , 17 , 18^

These models make it possible to explain several phenomena that were observed in the real arterial system but not interpretable by the Windkessel model. These include a secondary pressure wave in diastole or late systole, and amplification of the pressure pulse from the proximal aorta to the distal muscular arteries, explaining why arterial stiffness increases central pulse pressure and systolic arterial pressure (SAP). In young individuals and adults with healthy arterial aging, reflected waves generate retrograde waves, which must be superimposed, elevating the pressure during diastole, rather than systole, increasing coronary perfusion.^7 , 13 , 14 , 18 , 19^

Wave reflections originate in various locations, including bifurcations of conducting arteries and small muscular arteries. Vasoconstriction results in reflection points close to the heart, leading to early aortic wave reflections. The moment when wave reflections arrive at the proximal aorta depends on the PWV of conduit arteries. In addition, increased arterial stiffness, observed in older or hypertensive individuals, promotes an earlier arrival of the reflected wave, which travels rapidly along the arterial tree. Thus, both small and large arteries contribute to early wave reflection, which returns early in systole and superimposes on the forward wave. This process causes an increase in systolic blood pressure (SBP) and reductions in diastolic fluctuations and blood pressure ( Figure 1 ).

**Figure 1:** Arterial stiffness in large arteries. In a young health individual, a compliant aorta (left): 1) protects excess pulsatility caused by the intermittent left ventricular ejection and 2) exhibits a slow pulse wave velocity (PWV), allowing that reflected waves arrive to the heart during diastole, increasing diastolic coronary perfusion pressure but not after-load. Factors like aging and lifestyle increase aortic wall stiffness, leading to adverse hemodynamic consequences. Aortic stiffness leads to a rise in aortic root impedance, with consequent increase in forward wave amplitude and earlier arrival of the reflected waves to the heart. These hemodynamic changes result in adverse patterns of pulsatile load to the left ventricle during systole and a reduction in perfusion reserve (even in the absence of epicardial coronary disease). This inverse hemodynamic pattern also causes excess pulsatility in the aorta, which is preferably transferred to low-resistance beds, such as the kidney, placenta, and brain. In these organs, microvascular pressure is more directly associated with fluctuations in aortic pressure. AIx@75: augmentation index adjusted at heart rate. Source: the authors.

A pressure wave that propagates along a viscoelastic tube with numerous branches is progressively amplified from the central conduit towards distal arteries due to wave reflections and higher PWV in a stiffer peripheral artery. As a result, the amplitude of the pressure wave is higher in peripheral arteries than in central arteries, the so-called “amplification phenomenon”.^7 , 12 , 14 , 17 , 19 , 21^

## Non-Invasive Methods For The Assessment Of Arterial Stiffness

Arterial stiffness can be assessed at systemic, local, and regional levels. While systemic assessment of arterial stiffness can only be performed by models of the circulation, regional and local arterial stiffness can be measured directly, by non-invasive methods, with the advantage that the parameters used in these analyses are strongly linked to wall stiffness ( Table 1 ).^7 , 14 , 18 , 19^

**Table 1:** Device and methods used for determining regional, local, and systemic arterial stiffness and wave reflections

Year of first publication	Device	Method	Measurement site	Predictive value for CV events (year of first publication) clinical application	Easy clinical application
**Regional arterial stiffness**					
1984a	Complior®	Mechanotransducer	Aorta, cfPWVb	Yes (1999)	++
1990a	Sphygmocor®	Tonometer	Aorta, cfPWVb	Yes (2011)	++
1991	WallTrack®	Echotracking	Aorta, cfPWVb	No	+
1994	QKD	ECG +	Aorta, cfPWVb	Yes (2005)	++
1997a	Cardiovasc. Eng. Inc®	Tonometer	Aorta, cfPWVb	Yes (2010)	+
2002	Artlab®	Echotracking	Aorta, cfPWVb	No	++
2002	Sistema de ultrassom	Doppler probes	Aorta, cfPWVb	Yes (2002)	+
2002	Omron VP-1000®	Pressure cuff	Aorta, baPWVb	Yes (2005)	+++
2007	CAVI-Vasera®	ECG + Pressure cuff	Aorta, ctPWVb	Yes (2014)	+++
2008	Arteriograph®	Brachial pressure cuff	Aorta, aaPWVb	Yes (2013)	++
2009	RMN, ArtFun®	Magnetic resonance imaging	Aorta, aaPWVb	Yes (2014)	+
2010	Mobil-O-Graph®	Brachial pressure cuff	Aorta, cfPWVc	No	++
2010	Ultrafast®	Echography	Common carotid	No	–
2013	pOpmetre®	Plethysmography	Aorta, dpPWVb	No	+++
2017	Withings®	Ballistocardiography	Aorta	No	+++
**Local arterial stiffness**					
1991	WallTrack®	Echotracking	CCAd, CFA^d^, BA^d^.	No	+
1992	NIUS®	Echotracking	RA^d^	No	+/–
2002	Artlab®, Mylab®	Echotracking	CCAd, CFA, BA	Yes (2014)	++
2017	Ultrasound systems	Echography	CCAd, CFA, BA	No	+
2009	RMN, ArtFun®	Magnetic resonance imaging	AA^d^, DA^d^	No	+
**Systemic arterial stiffness**					
1989	Area method	Diastolic decay		No	+/–
1995	HDI PW CR-2000®	Modified Windkessel		No	+
1997a	Cardiovasc. Eng. Inc®	Tonometry/Doppler/ Echo		Yes (2010)	+/–
2009	MRI, ArtFun®	Magnetic resonance imaging	AA, DA	No	+

*^a^Device used in pioneer epidemiological studies that describe the predictive value of aortic stiffness for cardiovascular events;^b^PWV: pulse wave velocity, cf: carotid-femoral, ba: brachial-ankle, ca: cardiac-ankle, aa: aortic arch, ft: finger-toe.^c^Estimated (not measured).^d^All superficial arteries, including those particularly mentioned; Ao: aorta, CCA: common carotid artery, CFA: common femoral artery, BA: brachial artery, RA: radial artery, AA: ascendent aorta, DA: descendent aorta. Source: Adapted from Laurent at al. (2019, p. 143-144)*

## Regional determination of arterial stiffness

The aorta is the main vessels used for determination of regional arterial stiffness, since the thoracic and abdominal aorta are the main “cushions” of the arterial tree, and the aortic PWV is an independent predictor of cardiovascular outcomes.^22 - 32^

The carotid-femoral PWV (cfPWV) is the gold-standard, non-invasive method for arterial stiffness determination. Several studies using cfPWV measures have shown that arterial stiffness is related to cardiovascular events.^7 , 14 , 19 , 29 , 30 , 33^ cfPWV is determined by transcutaneous measures (tonometry) using the “foot-to-foot” velocity method in right carotid and femoral arteries ( Figure 2 ). The “foot” of the wave is defined at the end of diastole, when the sharp rise of the wavefront begins. cfPWV (m/s) is calculated by the formula cfPWV (m/s) = *D* (meters) /∆ *t* (seconds). ( *D* ) can be calculated as the (1) distance between two sites (carotid and femoral artery); (2) by subtracting the distance between the carotid site and manubrium sterni from total distance; (3) subtracting the distance between the carotid site and manubrium sterni from the distance between the manubrium sterni and the femoral site. Of all currently used measures, the 80% of the distance from the common carotid to the common femoral artery has been shown to be the most accurate.^3 , 13 , 14 , 19 , 32 , 33^

**Figure 2:** Measurement of carotid-femoral pulse wave velocity with the foot-to- foot method. Measurement of carotid-femoral pulse wave velocity with the foot-to-foot method. The waveforms are usually obtained transcutaneously, at the right common carotid artery and the right femoral artery. The time delay (Δt, or transit time) is measured between the feet of the two waveforms (Figure 1). The distance (ΔL) covered by the waves is usually the surface distance between two recording sites, i.e., the common carotid and the femoral artery. Pulse wave velocity (PWV) is calculated as PWV = 0.8 x ΔL (m)/Δt (s). Source: the authors.

The determination of cfPWV by tonometry has limitations, such as: a) the precise registration of the femoral pressure waveform may be difficult in patients with metabolic syndrome, obesity, and peripheral arterial disease; b) stenosis of the aorta, iliac artery, or proximal femoral artery may attenuate and halt pulse wave progression; and c) abdominal obesity, particularly in men, and large bust size in women can affect the accuracy of distance measurements.^3 , 13 , 14 , 19 , 32 , 33^

Therefore, the analysis of PWV from a unique site simplifies the measurement. Several devices have been developed to estimate the PWV in a given arterial pathway from the analysis of brachial pressure wave using an arm cuff. These methods include the determination of the time difference between onset time of electrocardiogram Q wave and onset time of Korotkoff sounds. Arteriograph® provides a single-point estimate of the PWV using a brachial cuff and supra-systolic oscillometry. The Mobil-O-Graph® (Brasil, Dyna Mapa AOP®) uses oscillometric registries obtained by three measurements of the brachial pressure waveform, at mean blood pressure (C1 calibration) or DBP (C2 calibration) to compose the pulse wave by the transfer function (ARCSolver® algorithm). In this last method, both age and blood pressure are used to refine the estimates of PWV.^13 , 14 , 19 , 32 , 33^

Reference values for cfPWV (tonometry) have been established for healthy individuals and those with cardiovascular risk factors from European countries.^34^ Also, reference values for the oscillometric method, of central systolic blood pressure (cSBP), aortic augmentation index (AIx) and PWV for individuals with and without cardiovascular risk factors have been established for the Brazilian population ( Table 2 ).^35^

**Table 2:** Reference values for central blood pressure, pulse wave velocity, and aortic augmentation index (AIx) for men and women, with and without cardiovascular risk factors

Age groups	Without cardiovascular risk factors		With cardiovascular risk factors	

	Women	Men	Women	Men
**cSBP**				
<30 years	101 (90; 93; 113; 119)	113 (104; 109; 120; 123)	118 (102; 109; 127; 131)	123 (107; 114; 132; 144)
30-39 years	109 (96; 102; 117; 123)	114 (102; 110; 121; 127)	120 (102; 110; 130; 143)	125 (108; 116; 133; 141)
40-49 years	110 (99; 103; 117; 122)	116 (102; 109; 122; 126)	121 (104; 112; 134; 146)	123 (108; 115; 131; 141)
50-59 years	110 (97; 104; 120; 124)	112 (100; 106; 1 18; 124)	124 (106; 114; 135; 146)	124 (105; 114; 134; 144)
60-69 years	114 (100; 105; 120; 125)	112 (96; 101; 120; 127)	127 (105; 115; 141; 154)	123 (103; 112; 136; 149)
70+ years	113 (100; 103; 121; 126)	116 (94; 104; 125; 129)	131 (108; 118; 146; 165)	125 (102; 111; 140; 156)
**cDBP**				
<30 years	73 (60; 66; 77; 85)	76 (66; 71; 82; 87)	82 (68; 73; 90; 97)	83 (72; 77; 93; 100)
30-39 years	77 (67; 71; 83; 88)	80 (7 1; 75; 85; 88)	86 (71; 77; 95; 105)	88 (75; 80; 96; 103)
40-49 years	79 (67; 73; 84; 88)	81 (74; 77; 86; 89)	86 (71; 78; 94; 103)	90 (75; 82; 97; 104)
50-59 years	76 (64; 70; 82; 85)	82 (70; 77; 86; 88)	84 (71; 77; 92; 100)	88 (75; 80; 97; 103)
60-69 years	76 (66; 71; 81; 87)	80 (68; 72; 83; 87)	81 (67; 74; 90; 98)	85 (71; 77; 93; 101)
70+ years	76 (60; 70; 79; 83)	79 (60; 70; 84; 90)	81 (66; 72; 89; 97)	82 (68; 74; 91; 98)
**cPP**				
<30 years	29 (23; 27; 37; 43)	36 (26; 32; 43; 53)	34 (24; 28; 41; 48)	38 (26; 31; 46; 52)
30-39 years	30 (22; 26; 37; 44)	35 (25; 29; 42; 50)	34 (24; 28; 38; 46)	36 (25; 31; 41; 48)
40-49 years	31 (22; 27; 36; 42)	32 (25; 28; 38; 45)	35 (25; 29; 43; 53)	33 (23; 28; 37; 46)
50-59 years	34 (25; 28; 42; 49)	30 (25; 27; 35; 42)	39 (28; 32; 47; 58)	34 (25; 28; 41; 49)
60-69 years	35 (28; 31; 43; 52)	31 (24; 28; 36; 49)	44 (30; 36; 55; 66)	37 (25; 31; 46; 58)
70+ years	39 (28; 34; 45; 52)	37 (19; 27; 41; 51)	50 (33; 41; 63; 77)	42 (28; 34; 52; 66)
**PWV**				
<30 years	4.9 (4.4;.4.5; 5.0; 5.3)	5.2 (4.9; 5.1; 5.4; 5.7)	5.3 (4.7; 5.0; 5.6; 6.0)	5.5 (5.0; 5.3; 5.8; 6.3)
30-39 years	5.4 (5.0; 5.2; 5.8; 6.1)	5.7 (5.3; 5.5; 5.9; 6.1)	5.8 (5.3; 5.5; 6.2; 6.7)	6.1 (5.5; 5.8; 6.4; 6.7)
40-49 years	6.4 (5.7; 6.0; 6.7; 6.9)	6.5 (5.9; 6.2; 6.8; 7.0)	6.8 (6.0; 6.4; 7.2; 7.7)	6.8 (6.2; 6.4; 7.1; 7.5)
50-59 years	7.5 (6.7; 7.0; 7.8; 8.2)	7.4 (6.9; 7.2; 7.9; 8.0)	7.9 (7.1; 7.5; 8.3; 8.8)	7.9 (7.1; 7.5; 8.3; 8.7)
60-69 years	8.9 (8.1; 8.5; 9.2; 9.4)	8.9 (8.2; 8.6; 9.1; 9.6)	9.3 (8.4; 8.8; 9.8; 10;4)	9.2 (8.4; 8.7; 9.7;10.2)
70+ years	11.3 (10.2;10.4;12.5; 13;2)	11.0 (10.1; 10.6; 11.6; 12.3)	11.8 (10.2; 10.8; 12.9; 14.0)	11.2 (9.9; 10.4; 12.1; 13.2)
**AIx**				
<30 years	20 (11; 13; 27; 33)	16 (4; 10; 23; 27)	28 (11; 20; 34; 38)	16 (2; 8;23; 30)
30-39 years	22 (12; 16; 28; 34)	14 (1; 7; 18; 24)	26 (11; 18; 32; 37)	15 (3; 9; 2 1; 27)
40-49 years	23 (9; 15; 29; 35)	15 (0; 6; 21; 25)	25 (10; 17; 34; 38)	15 (2; 8; 23; 30)
50-59 years	22 (7; 12; 33; 39)	12 (2; 4; 19; 22)	24 (8; 14; 33; 39)	15 (3; 7; 24; 32)
60-69 years	23 (9; 1 4; 34; 42)	17 (1; 5; 27; 43)	28 (11; 18; 37; 44)	1 7 (3;9; 26; 34)
70+ years	28 (11; 20; 39; 42)	22 (5; 10; 33; 41)	33 (17; 25; 42; 48)	22 (4; 12; 31; 41)

cSBP: central systolic blood pressure; cDBP: central diastolic blood pressure; cPP: central pulse pressure; PVW: pulse wave velocity; AIx: augmentation index. * Values expressed as 50º (10º. 25º. 75º. 90º) percentage points. †Number of women and men; without CVRF: <30 years (n=50 and 80): 30-39 years (n=134 and 70): 40-49 years (n=114 and 55): 50-59 years (n=121e 67): 60-69 year (n=80 e 38): 70+ years (n=32 e 26). ‡ Number of women and men with CVRF: <30 years (n=94 and 152): 30-39 years (n=240 and 297): 40-49 years (n=418 and 385): 50-59 years (n=827 and 638); 60-69 years (n= 919 and 561): 70+ years (n=671 and 430). § CVRF: cardiovascular risk factors. Source: Adapted from Paiva et al. (2020).

Despite the importance of PWV estimates in the prediction of cardiovascular events and risk stratification, the method is underused in clinical practice. A European group has proposed a clinical score, the SAGE, to identify patients with priority for PWV estimation, based on easily available variables: systolic blood pressure (S), age (A), fasting plasma glucose (G), and estimated glomerular filtration rate (E) (using the CKD-EPI).^36^ The score was applied in the Brazilian population using the oscillometric method and identified that hypertensive patients with SAGE ≥ 8 should be referred for arterial stiffness analysis, due to the high risk of increased PWV.^36 - 38^

### Assessment of local arterial stiffness

Local arterial stiffness can be determined by carotid ultrasound using the high-resolution echo-tracking. The method is highly accurate to determine diameter at diastole and stroke changes in diameter when compared with the classical analysis with other video-image systems. Chest nuclear resonance allows the combined determination of both structure and function of the heart and the aorta, with undoubted accuracy, but at the expense of lower spatial and temporal resolution. However, most of pathophysiological and pharmacological studies used echotracking techniques.^14 , 19 , 32 , 33^

### Systemic arterial stiffness

Method based on an electrical circuit using the modified Windkessel model, developed to determine the proximal capacitive compliance and distal oscillatory compliance. In addition, systemic arterial compliance can be determined using the “area method”, which requires the measurement of the aortic blood flow (velocity obtained from the suprasternal notch) and motor pressure associated with applanation tonometry on the right common carotid artery. Theoretical, technical, and practical limitations make the general application of this method in the clinical setting difficult.^14 , 19 , 33^

### Central blood pressure

Arterial pressure waveform should be analyzed at the central level (ascending aorta) as it represents the load imposed on the heart, kidney and arterial wall. The most widely used approach is radial artery tonometry, followed by application of a transfer function (SphygmoCor, Atcor, Sydney Australia) to calculate the aortic pressure waveform. The radial artery is sustained by bone tissue, which makes applanation easier.^7 , 14 , 19 , 32 , 33^

Aortic waveform can be estimated by common carotid artery tonometry, which requires more technical knowledge but does not require the transfer function, as the artery sites are very close, and the waveforms are similar. New methods have been developed to estimate central arterial pressure using the second systolic radial or brachial blood pressure peak. External calibration is necessary, made with brachial SBP and DBP to calibrate the radial artery tonometry, and with mean blood pressure and radial DBP to calibrate the aorta or carotid waveform.^7 , 14 , 19 , 32 , 33^

The pressure wave is composed by the wavefront, generated by ventricular contraction, and the retrograde wave, generated by wave reflected at bifurcation points. In elastic vessels, PWV is low and the reflected wave travels back towards the aorta root during diastole. In the presence of arterial stiffness, PWV increases, and the reflected wave returns early, adding “augmentation” during systole. This phenomenon can be quantified by the AIx, *i.e* ., the difference between the first and the second systolic peak (P2 – P1), in percentage ( Figure 3 ). Age and PWV are the main determinants of AIx.^7 , 14 , 19 , 32 , 33^

**Figure 3:** Carotid pressure waveform recorded by applanation tonometry. The wave reflection phenomenon can be quantified by the augmentation index (AIx), defined as the difference between the first (P1) and the second (P2) systolic peaks (P2 - P1 = AP, i.e., augmentation pressure), expressed as a percentage of pulse pressure (PP), AIx = AP / PP. Source: the authors.

In peripheral arteries, pressure wave amplitude is greater than in central arteries due to the amplification phenomenon; thus, peripheral SBP and brachial pulse pressure overestimate SBP and central pulse values in young individuals.^39^ Pulse wave should be analyzed through central pulse pressure (cPP), central SBP and the AIx.^14 , 19 , 32 , 33^ These parameters are independent predictors of all-cause mortality and cardiovascular events.^41 , 42^

Reference values for cSBP and AIx were defined for the European population^43^ by tonometry and for the Brazilian population by the oscillometric method^35^ ( Table 2 ).

cSBP, cPP, AIx and PWV cannot be used indistinctly as indicators of arterial stiffness, since they are different determinants. cSBP, cPP and AIx depend on PWV, amplitude of the reflected wave, the reflection point, and the ejection fraction duration and pattern, especially those related to changes in heart rate (HR) and ventricular contractility. Pathophysiological and pharmacological conditions can affect both cPP and AIx without affecting the aortic PWV, suggesting a predominant effect of the reflected wave, HR and ventricular ejection, and no change in aortic stiffness. The influence of age is greater on AIx than on PWV before the age of 50 and greater on PWV than AIx after this age. Therefore, while PWV is a direct measure of arterial stiffness, cSBP and AIx are indirect measures.^7 , 14 , 19 , 32 , 33^

## Arterial stiffness and the cardiovascular continuum

The classical description of the “cardiovascular continuum”, published by Dzau et al. (2006)^44^ reports the progression of CVD ( Figure 4 ) founded on the atherosclerosis process, which is initiated with the exposure to risk factors (hypertension, diabetes, dyslipidemia, smoking, and obesity), progressing in stages that culminate in the obstruction of coronary arteries, ischemia, and myocardial infarction, end-stage heart disease, heart failure, and death. While this model highlights gene-related pathophysiological aspects, molecules, chemical processes and intracellular mechanisms associated with atherosclerosis, it ignores contributions from cardiovascular aging, derived from physical and mechanical changes of vascular structures.^3 , 45 , 46^

**Figure 4:** Comparison between classic cardiovascular continuum (A) and aging cardiovascular continuum (B). Source: Barroso; Barbosa; Mota-Gomes, 2020.

In 2010, a novel model was proposed – the Cardiovascular Aging Continuum^46^ ( Figure 4 ) – based on the arteriosclerosis process. It initiates with arterial aging, progresses to end-stage cardiac, cerebral and renal microvascular disease, disability and death.^3 , 7 , 46^

This new approach highlights the progressive aorta degeneration with deleterious effect to the target organs. It extends the considerations of either obstructive or ischemic arterial disease to the progressive aging-related stiffening of elastic arteries, manifested as increased PWV and AIx.^3 , 7 , 46^ A 1m/s increase in aortic PWV was associated with a 15% increment in cardiovascular mortality and all-cause mortality.^29^ Analysis of cSBP, PWV and AIx revealed that they were better predictors of cardiovascular risk and mortality than peripheral blood pressure.^29 , 30^

The cardiovascular aging continuum is divided into four stages, as follows ( Figure 5 ).^3 , 46^

**Figure 5:** Association between classic cardiovascular continuum and aging cardiovascular continuum. LV: left ventricular; CAD: coronary artery disease; VE: ventricular enlargement. Source: Barroso; Barbosa; Mota-Gomes, 2020.

- Stage 1: The heartbeats lead to fracture and fraying of elastic lamellae, with consequent aortic dilation and transfer of mechanical stress to collagen fibers, responsible for arterial stiffness.^3 , 46^
- Stage 2: Aortic stiffening leads to elevation of SBP, caused by stiffening of proximal aorta and an earlier return of the reflected wave during systole. Consequently, there is increase in ventricular afterload, left ventricular hypertrophy, increased myocardial oxygen consumption and reduction in coronary perfusion.^3 , 46^
- Stage 3: Intermittent cardiac contractions transfer the pulsatile flow to the stiffened aorta (decreased cushioning capacity) and extend peripherally into the microvasculature, with consequent increase in shear stress, particularly in small arteries of organs with high resting blood flow and low microvascular resistance (the brain, kidney, testicles, liver and placenta).^3 , 46^
- Stage 4: contractions of the hypertrophied heart occur slowly, so that systole duration is increased, and diastole duration is decrease in any HR. These changes affect coronary blood flow, which fails to supply the demand as both aortic pressure during diastole and diastole period are decreased. The combination of higher supply and decreased coronary perfusion capacity predisposes to ischemia, regardless of coronary narrowing, which is aggravated in atherosclerosis. A vicious cycle is then established: ischemia causes prolongation of ventricular relaxation and ejection time, which, in turn, aggravates ischemia.^3 , 46^

Although the two “continuums” can be seen independently, they interact in the development of end-stage CVD. They share the same final pathways, that describe complications of myocardial ischemia and progression to end-stage heart disease, as consequence of arterial stiffening and narrowing. The two continuums are combined in Figure 5 to explain the deleterious effects of atherosclerotic disease and of aging, as these progress over years and culminate in the diseases of old age.^46^ Cardiac insufficiency is commonly associated with cerebral and renal microvascular disease, causing intellectual deterioration and renal failure.^46^

The brain requires a high blood supply and low arterial resistance, and is susceptible to pulsatile microvascular trauma and hypoperfusion, especially in white matter, which is less vascularized and less perfused than the grey matter. Changes in cerebral perfusion due to an increase in pulsatility lead to microvascular remodeling and low oxygenation, and progressive cognitive decline, dementia, subclinical infarction, and cerebrovascular accident.^7 , 47 - 49^

The kidney exhibits the highest flow rate and the lowest vascular resistance as compared to the other organs. For this reason, it is susceptible to trauma due to pulsatile flow, and consequent glomerular damage, albuminuria, and reduction of glomerular filtration rate. CKD also causes stiffening of large arteries because of an imbalance in bone mineral metabolism (increase in osteoprotegerin, fibroblast growth factor and inflammatory cytokines), and increased vascular calcification. Hyperactivity of the ANS and the RAAS reduces sodium elimination, contributing to arterial stiffening. In individuals with CKD, particularly diabetic patients, PWV increases. The stiffening of large arteries independently predicts a higher risk of cardiovascular events in CKD patients.^7 , 25 , 26^

Aging leads to increased vascular stiffening and changes in microcirculation, resulting in the decline of cardiac, cerebral, and renal function. It is possible that microvascular damage may be prevented and/or delayed with therapy aimed at reducing arterial stiffness and wave reflection.^7^

## Arterial aging and cardiovascular risk

Part of the residual cardiovascular risk in hypertensive patients has been related to the AVA process. The early detection allows a more effective cardiovascular protection. In the pathophysiology of CVD, there is a bidirectional interaction between AVA and hypertension.^1 , 10 , 45^

Classical risk factors are important for selecting, evaluating and guiding lifestyle habits and pharmacological therapy. However, the risk of CVD still represents a challenge; despite prevention and treatment efforts, there is still a need for new pathophysiological models for a better understanding of CV risk and of CVD treatment.^3 , 45 , 50^

It has been shown that target organ lesion, like LVH and increased microalbuminuria, represent the boundary between cardiovascular risk factors and cardiovascular events.^45^ Besides, arterial stiffness, increased PWV and increased cSBP are independent predictors of cardiovascular events.^29 , 30^ These are examples of an underlying pathological process, since an elevation in PWV can determine the degree of LVH by the increase in the arterial pulse wave reflection, CPP and after-load.^7 , 19^

Therefore, arterial stiffness is useful to guide clinical investigations in individuals at low and moderate cardiovascular risk.^1 , 10^ These parameters, considered as arterial “biomarkers”, can be better predictors than high-sensitive C reactive protein.^32 , 45^ The addition of PWV during risk classification improved risk prediction (13% for CVD in 10 years for intermediate risk).^30^ This information, when correctly accessed and used, prevent the misclassification of high-risk patients as low or moderate risk.^45 , 50^

## Perspectives

Vascular aging is responsible for the increase of residual cardiovascular risk and the global CVD burden. Further studies are needed for clinical validation of the cardiovascular outcomes, comparisons between different assessment methods and studies of therapeutical interventions mediated by researchers’ network on vascular aging. Continuous education and the wide use of technologies on preventive strategies should be encouraged, aimed at highlighting the role of vascular aging and integrating it into the clinical decision making.^3 , 50 , 51^

Science has attempted to improve the understanding and the clinical applicability of biomarkers able to early identify vascular damage. The objective is to improve accuracy in cardiovascular risk stratification in low-to-moderate risk individuals.^32^ Analysis of cSBP and arterial stiffness (PWV) is support by strong evidence for early detection of vascular damage, and identification and reclassification of individuals who were initially classified as low/intermediate risk and were later classified as high risk.^30 , 45^ In addition, a PWV ≥ 10m/s can be suggestive of subclinical target-organ damage, and the increase in cSBP is a predictor of arterial hypertension.^7 , 19 , 30 , 52^ It is possible that, as new evidence emerges in the context of hypertensive disease and CVD, this method becomes more reliable and safer to be incorporated into clinical practice, aiming to early identify vascular damage.^50^ In the realm of precision medicine, this approach allows a tailored clinical practice, with greater assertiveness in the decisions related to classification and treatment of CVD.^50^
